# SPOCK1 Is a Novel Transforming Growth Factor-β–Induced Myoepithelial Marker That Enhances Invasion and Correlates with Poor Prognosis in Breast Cancer

**DOI:** 10.1371/journal.pone.0162933

**Published:** 2016-09-14

**Authors:** Li-Ching Fan, Yung-Ming Jeng, Yueh-Tong Lu, Huang-Chun Lien

**Affiliations:** 1 Department of Pathology, National Taiwan University Hospital, Taipei, Taiwan; 2 Graduate Institute of Pathology, College of Medicine, National Taiwan University, Taipei, Taiwan; University of Navarra, SPAIN

## Abstract

In addition to contraction, myoepithelia have diverse paracrine effects, including a tumor suppression effect. However, certain myoepithelial markers have been shown to contribute to tumor progression. Transforming growth factor-β (TGF-β) is involved in the transdifferentiation of fibroblasts to contractile myofibroblasts. We investigated whether TGF-β can upregulate potential myoepithelial markers, which may have functional and clinicopathological significance in breast cancer. We found that TGF-β induced SPOCK1 expression in MCF10A, MCF12A, and M10 breast cells and demonstrated SPOCK1 as a novel myoepithelial marker that was immunolocalized within or beneath myoepithelia lining ductolobular units. A functional study showed that overexpression of SPOCK1 enhanced invasiveness in mammary immortalized and cancer cells. To further determine the biological significance of SPOCK1 in breast cancer, we investigated the expression of SPOCK1 in 478 invasive ductal carcinoma (IDC) cases through immunohistochemistry and correlated the expression with clinicopathological characteristics. SPOCK1 expression was significantly correlated with high pathological tumor size (P = 0.012), high histological grade (P = 0.013), the triple-negative phenotype (P = 0.022), and the basal-like phenotype (P = 0.026) and was correlated with a significantly poorer overall survival on univariate analysis (P = 0.001, log-rank test). Multivariate Cox regression analysis demonstrated that SPOCK1 expression maintained an independent poor prognostic factor of overall survival. Analysis of SPOCK1 expression on various non-IDC carcinoma subtypes showed an enrichment of SPOCK1 expression in metaplastic carcinoma, which is pathogenetically closely related to epithelial-mesenchymal transition (EMT). In conclusion, we identified SPOCK1 as a novel TGF-β–induced myoepithelial marker and further demonstrated that SPOCK1 enhanced invasion in breast cancer cells and correlated with poor prognosis in breast cancer clinical samples. The enrichment of SPOCK1 expression in metaplastic carcinoma and the correlation between SPOCK1 expression and high histological grading and basal-like phenotypes in IDC evidence an association between SPOCK1 and EMT.

## Introduction

The mammary epithelium is composed of 2 cell layers, the inner luminal cells and the outer myoepithelial cells (MECs). MECs have dual epithelial and smooth muscle phenotype. The function of MECs has traditionally been considered to be restricted to milk ejection during lactation, however, accumulating evidence has revealed that in addition to contraction, MECs have diverse paracrine effects related to epithelial differentiation and extracellular matrix formation [1]. Although a tumor suppressor role has been implicated in MECs [1,2], studies have suggested that MECs may contribute to the tumorigenesis of triple-negative breast tumors [1,3,4]. Moreover, a subset of breast tumors expressing MEC markers (e.g., CK5, caveolin 2, and secreted protein, acidic, cysteine-rich (SPARC)) are characterized by a particularly poor clinical outcome [5–7]. This indicates that certain MEC markers may have tumor progressive effects and thus correlate with poorer prognosis. Identification of such MEC markers may contribute to the understanding of MEC marker-related mammary pathogenesis.

Transforming growth factor-β (TGF-β) is a known mediator of tissue repair and wound healing [8,9]. In addition to its effect on extracellular matrix turnover, TGF-β is involved in the process of transdifferentiation of fibroblasts toward myofibroblasts during wound healing through the induction of a contractile phenotype and the upregulation of α-smooth muscle actin [10–12]. On the basis of the ability of TGF-β in the induction of contractile phenotype during fibroblast-myofibroblast transdifferentiation and on the dual epithelial and contractile phenotype in MECs, this study investigated whether TGF-β can upregulate potential MEC markers that can have functional and clinicopathological significance in breast cancer. Information derived would contribute to a better understanding of the role of TGF-β on breast cancer biology.

## Materials and Methods

### Cell culture

The MCF10A and MCF12A breast cell lines, obtained from The American Type Tissue Culture Collection, were maintained in DMEM/F12 medium (Life Technologies, Carlsbad, CA, USA) as previously described [13]. The H184B5F5/M10, MDA-MB231 and T47D cell lines were obtained from Bioresource Collection and Research Center (Hsinchu, Taiwan). The MDA-MB231 and T47D cell lines were maintained in DMEM medium supplemented with 10% FBS and the H184B5F5/M10 cell line was maintained in MEM medium supplemented with 10% FBS (Life Technologies). All cells were incubated at 37 °C in a humidified 5% CO_2_ atmosphere. For TGF-β1 induction, cells were treated with recombinant human TGF-β1 (R&D Systems, Minneapolis, MN, USA) at a concentration of 5 ng/mL for the indicated time before analysis.

### Microarray analysis

Total RNA for microarray analysis was prepared as previously described [14]. The microarray experiment and data analysis were done by Welgene Biotech (Taipei, Taiwan) using the Agilent Oligo Chip (Agilent SurePrint G3 Human V2 GE 8×60K Microarray, Agilent Technologies, USA). Microarrays were scanned by laser scanner and the microarray signal intensities were measured to identify gene expression differences and ratios of gene expression.

### Plasmid construction and transient transfection

The SPOCK1-expressing plasmid, pCDH-SPOCK1, was constructed by subcloning the SPOCK1 cDNA (GeneScript, Piscataway, NJ, USA) into the pCDH-CMV-MCS-EF1-GFP expression vector (System Biosciences, Mountain View, CA, USA). The plasmid was subsequently transfected into cells by using the Lipofectamine 3000 reagent (Invitrogen, Carlsbad, CA, USA).

### Reverse transcription–polymerase chain reaction (RT–PCR) and Western blot

Total RNA was reverse transcribed into cDNA and RT–PCR using standard protocols [14]. The primer sets used were as follows: SPOCK1 forward primer, 5’-GTTCTACTGGCAAAAGCCTCGC, SPOCK1 reverse primer, 5’-AGGTTCCGCAACTCCTTGTCTG, internal control S26 ribosomal protein forward primer, 5’-CCGTGCCTCCAAGATGACCAAAG, and S26 reverse primer, 5’-GTTCGGTCCTTGCGGGCTTCAC. Whole-cell lysates were made in RIPA buffer and subjected to SDS-PAGE, transferred onto a polyvinylidene difluoride membrane (Millipore, Billerica, MA, USA), and incubated with primary antibodies SPOCK1 (1:1000, HPA007450, Sigma-Aldrich, St. Louis, MO, USA) and GAPDH (ab9485, Abcam, Cambridge, MA, USA). The western blots were then incubated with horseradish peroxidase–conjugated secondary antibodies and further detected using an enhanced chemiluminescence kit (T-Pro Biotechnology, Taipei, Taiwan).

### In vitro invasion assay

The invasive capability of cells was examined using polycarbonate transwell filters containing 8-μm pores (Corning Coster, Cambridge, MA, USA). Cells (2 × 104) seeded in serum-free medium on the upper side of the chamber coated with Matrigel (BD Biosciences, Bedford, MA, USA). The cells were allowed to migrate toward the lower chamber containing media supplemented with 10% FBS. After 16-hours, cells on the lower side of the membrane were fixed, stained with crystal violent and then counted.

### Cell proliferation assay

Cell proliferation was measured using a 3-(4, 5-dimethylthiazol-2-yl)-2, 5-diphenyltetrazolium bromide (MTT) assay. Cells were counted and seeded in 96-well plates, and then incubated at 37°C in a humidified 5% CO2 atmosphere. Twenty microliters of MTT reagent (5 mg/ml, Sigma) was added to each well at the end of incubation, then 4 hours later the medium was discarded and dimethylsulfoxide (DMSO) was added to each well to dissolve the purple crystal. Then the absorbance at 570 nm was measured.

### Tumor sample and immunohistochemistry

Four hundred seventy-eight archives of breast invasive ductal carcinoma (IDC) paraffin tissue blocks from the Department of Pathology of National University (1993–2001) were arrayed in a high-density tissue array by using a 1-mm-diameter punch instrument, and the follow-up data of these patients were collected and used for survival analysis. Depending on tumor size, 2 to 4 cores were collected from each tumor case. Additional eighty-one cases of non-IDC breast carcinoma subtypes tissue blocks, including 35 invasive lobular carcinomas, 7 micropapillary carcinomas, 10 mucinous carcinomas, 8 invasive papillary carcinomas, 4 neuroendocrine carcinomas, 2 tubular carcinomas and 15 metaplastic breast carcinomas, were included for the analysis in breast carcinoma subtypes. Sections from primary breast cancer tissues were stained through immunohistochemistry as previously described [13]. The slides were incubated with a rabbit polyclonal antibody against human SPOCK1 (1:200, HPA007450, Sigma-Aldrich) and incubated with polymer-HRP reagent (Dako Cytomation, Glostrup, Denmark). Unequivocal staining in equal to or higher than 5% of tumour cells was considered positive. Negative control slides were processed without primary antibody and were included for each staining. The remaining antibodies used for determining potential TGF-β-induced MEC markers were POSTN (GTX100602, GeneTex, Inc., Irvine, CA, USA), CXCR7 (HPA049718, Sigma-Aldrich), IGFL2 (HPA059137, Sigma-Aldrich), PCDH9 (HPA015581, Sigma-Aldrich), PRRX2 (HPA026808, Sigma-Aldrich), FBN1 (HPA017759, Sigma-Aldrich), PLAT (HPA003412, Sigma-Aldrich), and NOV (HPA019684, Sigma-Aldrich). All slides were reviewed by 2 pathologists (HCL and YMJ). This study was approved by Institutional Review Board of National Taiwan University Hospital. Because the tumor blocks from the 478 IDC cases that were used in the survival analysis were collected before November 2003 and were used only for immunohistochemistry, the written informed consent requirements were waived based on the regulation of our Institutional Review Board. The consent for the tumor blocks from the 81 breast cancer subtypes cases were not obtained because all patient information was anonymised with all patient identifiers removed.

### Survival analysis

Statistical analyses were conducted using SPSS version 18.0 for Windows (SPSS Inc., Chicago, IL, USA). The chi-square test was performed to analyze the correlation between SPOCK1 expression and clinicopathological parameters. The cumulative overall survival was calculated using the Kaplan-Meier method, and the log-rank test was used to analyze differences in the survival times. Univariate and multivariate survival with calculation of hazard ratios (HR) were performed using the Cox regression model. Statistical significance was set as P < 0.05.

## Results

### Identification of SPOCK1 as a novel TGF-β–induced myoepithelial marker

**Fig 1:**
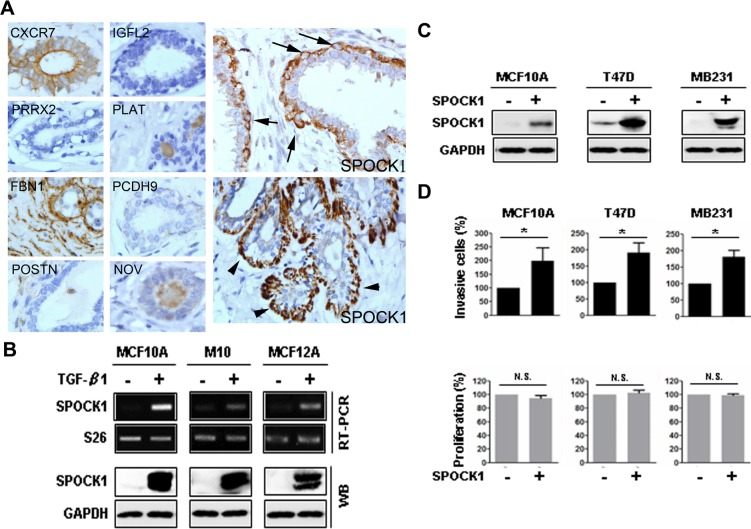
SPOCK1 was a TGF-β-induced myoepithelial marker and SPOCK1 overexpression enhanced invasiveness. (A) Immunohistochemistry demonstrated that SPOCK1 was the only myoepithelial marker among the evaluated TGF-β–upregulated gene products in MCF10A cells. CXCR7 staining was observed in luminal epithelia but not myoepithelia whereas FBN1 staining was observed diffusely in the stroma but not in the epithelia. IGFL2, PRRX2, PLAT, PCDH9, POSTN and NOV staining was not found in the ductolobular units (left and middle panels). By contrast, SPOCK1 was immunolocalized within (arrow) or beneath (arrowhead) the myoepithelia (right panel). (Magnification × 400). (B) Upregulation of SPOCK1 at mRNA levels (upper panels) and protein levels (lower panels) were observed in MCF10A, M10, and MCF12A cells, 3 days after treatment with TGF-β. S26 was used as an mRNA loading control and GAPDH was used as a protein loading control. (C) Western blotting was used to detect the expression of SPOCK1 in MCF10A, T47D, and MB231 cells 24 h after transient transfection with pCDH-SPOCK1 or control vector pCDH. (D) The invasive capability and proliferation were measured in the cells shown in (C). Data from invasion assay are shown as the mean ± SD of 3 fields. Data from MTT assay are shown as mean ± SD of 3 independent experiments. These results are presented as the percentage relative to their control cells (*, P < 0.05; N.S., nonsignificant).

To identify potential TGF-β-induced myoepithelial markers, we treated human mammary epithelial MCF10A cells with TGF-β. We initially focused on the top 30 TGF-β–upregulated genes (S1 Table). Of the 30 genes, we investigated protein expression of the genes for which antibodies suitable for immunohistochemistry of the gene products were available but for which information on the expression of the gene products, particularly regarding MEC, was not yet available (S1 Table). Among the 9 genes evaluated—namely POSTN, CXCR7, IGFL2, SPOCK1, PCDH9, PRRX2, FBN1, PLAT, and NOV—we found that SPOCK1 (SPARC/osteonectin, CWCV and kazal-like domains proteoglycan 1) was the only myoepithelial marker that was immunolocalized within or beneath the MECs (Fig 1A). The upregulation of SPOCK1 at RNA and protein levels by TGF-β were also observed in 2 other mammary epithelial cells, MCF12A and M10 cells, in addition to MCF10A cells (Fig 1B), which confirmed SPOCK1 as a novel TGF-β-induced myoepithelial marker.

### SPOCK1 enhanced invasion in immortalized breast epithelial cell and breast cancer cells

To test the hypothesis that certain MEC markers may have tumor progressive effect and confer adverse prognosis in breast cancer, we characterized the functional role of SPOCK1. We overexpressed SPOCK1 through transient transfection in MCF10A cells and 2 breast cancer cell lines, T47D and MB231, and performed invasion assay. SPOCK1 expression at the RNA and protein levels were confirmed through RT–PCR and Western blotting, respectively (Fig 1C). Notably, overexpression of SPOCK1 in MCF10A cells significantly promoted the invasive capabilities, compared with control cells. This observation was further confirmed in T47D and MB231 cells (Fig 1D). However, overexpression of SPOCK1 had no significant effects on cell proliferation (Fig 1D), indicating that the difference in invasion was not caused by proliferation. These results demonstrate a role for SPOCK1 in mammary cell invasion.

### Clinicopathological significance of SPOCK1 expression in human breast cancer

**Fig 2:**
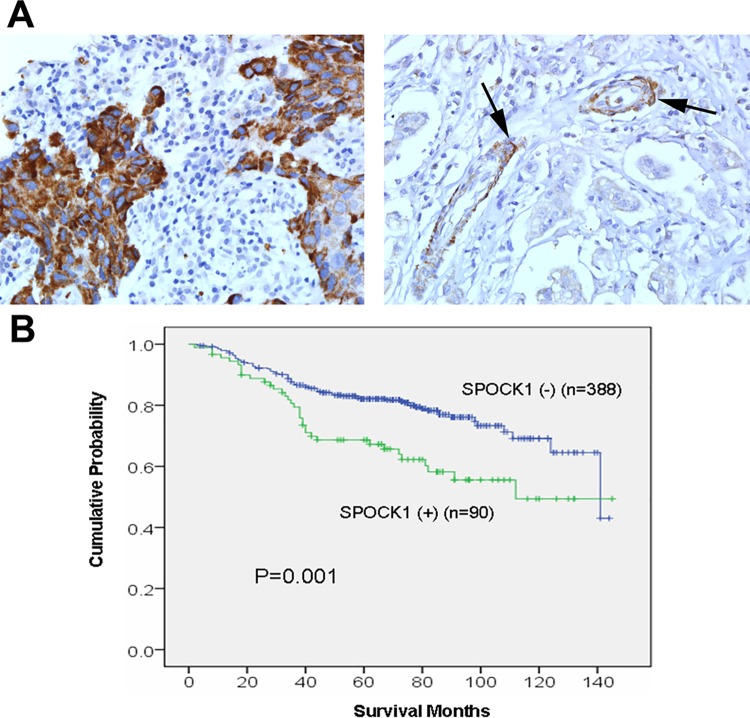
SPOCK1 expression and Kaplan–Meier analysis of overall survival in IDC patients. (A) Representative examples of IDC which were positive (left) and negative (right) for SPOCK1 expression. Note that the SPOCK1 expression in vascular smooth muscle wall (arrow) serve as internal positive control (magnification ×400). (B) SPOCK1 expression in IDCs was significantly associated with decreased overall survival (P = 0.001, log-rank test).

To investigate the clinicopathological significance of SPOCK1 expression, we analyzed the association between SPOCK1 expression and clinicopathological parameters in 478 cases of IDC with available clinicopathological information (Table 1 and Fig 2A). In normal breast, SPOCK1 was expressed consistently within or beneath the MECs lining the ductolobular units and also in the smooth muscle vessel walls. Luminal cells, by contrast, showed negative staining. SPOCK1 expression was observed in 18.8% (90/478) of IDC specimens, and the expression was positively correlated with a high pathological tumor size (P = 0.012), a high histological grade (P = 0.013), the triple-negative phenotype (ER^**−**^, PR^**−**^, and HER2^**−**^; P = 0.022), and the basal-like phenotype (ER^**−**^, PR^**−**^, HER2^**−**^ and CK5/6^**+**^; P = 0.026). To investigate whether SPOCK1 expression demonstrated any prognostic impact, we analyzed the association between SPOCK1 expression and overall survival in IDC cases with available overall survival data. In the Kaplan–Meier survival curve for overall survival, a statistically significant poorer prognosis was observed in the SPOCK1-positive patients compared with the SPOCK1-negative patients (P = 0.001, log-rank test; Fig 2B). A further multivariate Cox regression analysis demonstrated that SPOCK1 expression status maintained prognostic significance with respect to overall survival after adjustment, with a hazard ratio of 1.565 (95% CI = 1.045 to 2.343, P = 0.030; Table 2).

**Table 1:** Association of SPOCK1 expression with clinicopathological factors for 478 cases of IDC.

Clinicopathological variables		SPOCK1 expression			Chi-square test	
		Negative	Positive	Total^a^	χ^2^	P
		n (%)	n (%)			
Age (years)					-0.020	0.662
	≦50	197 (80.4)	48 (19.6)	245		
	>50	191 (82.0)	42 (18.0)	233		
Pathological tumor size (cm)					0.106	0.012*
	T1	125 (89.3)	15 (10.7)	140		
	T2	215 (77.3)	63 (22.7)	278		
	T3 and T4	47 (79.7)	12 (20.3)	59		
Pathological lymph node status					0.027	0.558
	Negative	168 (82.4)	36 (17.6)	204		
	Positive	215 (80.2)	53 (19.8)	268		
Pathological tumor stage					0.104	0.079
	I	78 (87.6)	11 (12.4)	89		
	II	183 (82.1)	40 (17.9)	223		
	III	122 (76.3)	38 (23.8)	160		
SBR grade					0.105	0.013*
	I	118 (83.7)	23 (16.3)	141		
	II	189 (84.4)	35 (15.6)	224		
	III	81 (71.7)	32 (28.3)	113		
Estrogen receptor					-0.143	0.002*
	Negative	122 (73.5)	44 (26.5)	166		
	Positive	266 (85.3)	46 (14.7)	312		
Progesteron receptor					-0.051	0.261
	Negative	135 (78.5)	37 (21.50)	172		
	Positive	253 (82.7)	53 (17.3)	306		
HER2^b^					0.058	0.205
	Negative	316 (82.3)	68 (17.7)	384		
	Positive	72 (76.6)	22 (23.4)	94		
Positivity for triple negativity^c^					0.105	0.022*
	Negative	335 (82.9)	69 (17.1)	404		
	Positive	53 (71.6)	21 (28.4)	74		
Basal-like phenotype^d^					0.102	0.026*
	Negative	377 (82.0)	83 (18.0)	460		
	Positive	11 (61.1)	7 (38.9)	18		
Adjuvant CT and/or HT					0.068	0.152
	Negative	24 (92.3)	2 (7.7)	26		
	Positive	344 (81.1)	80 (18.9)	424		

SBR grade, Scarff–Bloom–Richardson grade; CT, chemotherapy; HR, hormone therapy

*Significance level was set at P < 0.05

^a^Numbers do not always add up to 478 because of the lack of tumor excision and/or lymph node sampling in some cases.

^b^HER2-positive: HER2: 3+/3+ (IHC) or 2+/3+ (IHC) and positive on HER2 FISH test. HER2-negative: HER2: 0–1+/3+ (IHC) or HER2: 2+/3+, but negative on HER-2/neu FISH test. A commercially available dual-color FISH kit for simultaneous evaluation of HER-2/neu gene and chromosome 17 copy number was used according to the manufacturer instructions (PathVysion™ HER-2 DNA Probe Kit, Vysis, Inc., Downers Grove, IL, USA).

^c^Positivity for triple negativity: ER^**−**^, PR^**−**^, and HER2^**−**^.

^d^Basal-like phenotype: ER^**−**^, PR^**−**^, HER2^**−**^, and CK5/6^+^ (>10% tumor cells).

**Table 2:** Cox regression model analysis of the clinicopathological variables regarding overall survival in IDC patients.

Clinicopathological variables	Overall survival			
	Univariate survival analysis		Multivariate survival analysis	
	Hazard ratio (95% CI)	P-value	Hazard ratio (95% CI)	P-value
Age (≦50 *vs*. >50 years)	1.076 (0.750–1.542)	0.691		
SBR grade (I *vs*. II/III)	2.374 (1.467–3.842)	<0.001	2.096 (1.271–3.456)	0.004
Involved lymph node (negative *vs*. positive)	2.129 (1.423–3.186)	<0.001	1.652 (1.061–2.571)	0.026
Tumor stage (I *vs*. II/III)	4.195 (1.953–9.010)	<0.001	2.445 (1.055–5.669)	0.037
Positivity for triple negativity (negative *vs*. positive)	2.127 (1.401–3.230)	<0.001	1.805 (1.181–2.760)	0.006
SPOCK1 expression (negative *vs*. positive)	1.938 (1.306–2.876)	0.001	1.565 (1.045–2.343)	0.030

### Expression of SPOCK1 in breast carcinoma subtypes

**Fig 3:**
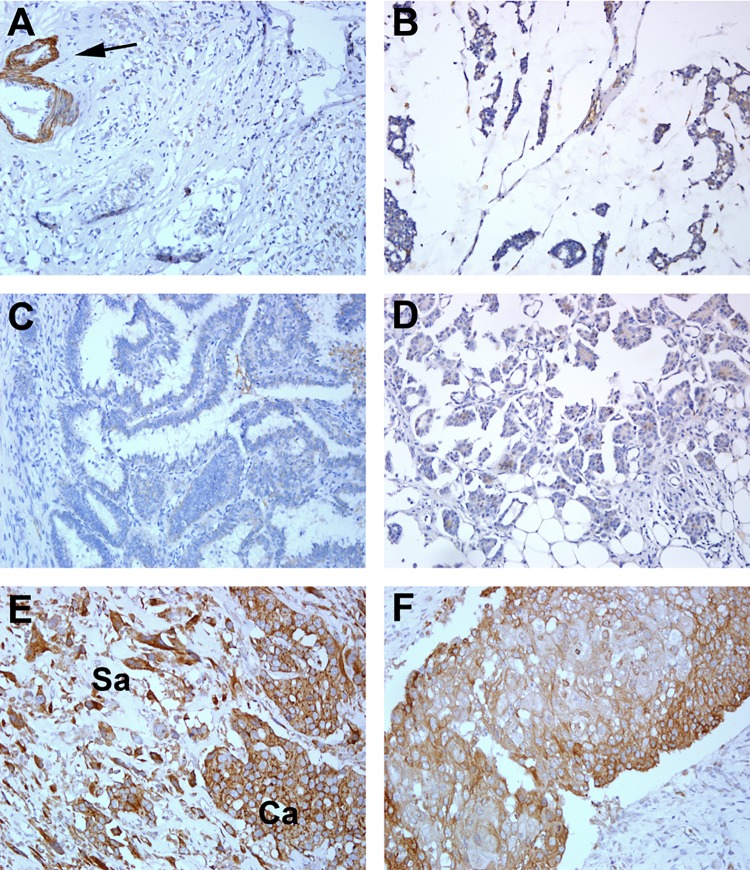
Immunohistochemical staining for SPOCK1 in various subtypes of breast carcinoma. SPOCK1 was not expressed in representative cases of invasive lobular carcinoma (A), mucinous carcinoma (B), invasive papillary carcinoma, (C) and invasive micropapillary carcinoma (D). SPOCK1 expression in vascular smooth muscle wall (arrow) served as internal positive control. Two representative SPOCK1-positive cases of metaplastic carcinoma are shown (E and F). Intense SPOCK1 expression was observed in both carcinomatous (Ca) and sarcomatous (Sa) elements in a representative biphasic metaplastic carcinoma (E). SPOCK1 was strongly stained in one representative monophasic epithelial metaplastic carcinoma (squamous metaplasia) (F). (Magnification ×200).

To determine the SPOCK1 expression status in non-IDC breast carcinoma subtypes, we performed immunohistochemistry for SPOCK1 in 81 breast carcinomas of different histological subtypes; representative figures are shown in Fig 3. Compared with the expression in 18.8% of IDC cases, SPOCK1 expression was rarely observed in cases of invasive lobular carcinoma (2/35), micropapillary carcinoma (1/7), mucinous carcinoma (0/10), invasive papillary carcinoma (0/8), invasive neuroendocrine carcinoma (0/4), and tubular carcinoma (0/2) (Table 3). In sharp contrast to the aforementioned carcinoma subtypes, over half of the metaplastic breast carcinoma (MBC; 10/15, 66.7%) were strongly positive for SPOCK1 (Table 3). These 15 MBC cases included 4 cases in the original cohort and 11 additionally included cases because of the high expression of SPOCK1 in MBC (3/5) in the original cohort. In SPOCK1-positive biphasic MBC cases, staining was observed in both sarcomatous and carcinomatous components.

**Table 3:** SPOCK1 protein expression in non-IDC breast carcinoma subtypes.

Carcinoma subtypes	SPOCK1 expression		
	Positive	Negative	Total
	n (%)	n (%)	n (%)
Invasive lobular carcinoma	2 (5.7)	34 (94.3)	35 (100)
Mucinous carcinoma	0 (0)	10 (100)	10 (100)
Papillary carcinoma	0 (0)	8 (100)	8 (100)
Micropapillary carcinoma	1 (14.2)	6 (85.8)	7 (100)
Tubular carcinoma	0 (0)	2 (100)	2 (100)
Neuroendocrine carcinoma	0 (0)	4 (100)	4 (100)
Metaplastic carcinoma	10 (66.7)	5 (33.3)	15 (100)

## Discussion

SPOCK1, also known as testican-1, is a heparin/chondroitin-sulfate–bearing proteoglycan originally identified in human testicular seminal plasma [15–17]. It belongs to a matricellular protein family named SPARC. The SPARC family of proteins consists of SPARC (osteonectin), Hevin (SPARC-like protein 1), secreted modular calcium binding protein (SMOC) 1 and 2, testican-1, 2 and 3, and follistatin like protein 1 [18]. Members of the SPARC family share a follistatin-like domain and an extracellular calcium binding E-F hand motif, regulate extracellular matrix assembly and deposition, and modulate growth factor signaling pathways [18,19]. In the present study, we identified SPOCK1 as a novel MEC marker. Interestingly, expression profiling of purified normal human luminal and myoepithelial breast cells has identified SPARC (osteonectin) as a novel myoepithelial marker [7]. These results illustrated that members of the calcium-binding SPARC family play a role in the function of mammary MEC. Another gene expression profiling study using serial analysis of gene expression to analyze freshly isolated uncultured luminal epithelial and myoepithelial cells reported that a high fraction (43%) of the genes differentially expressed in MEC encode secreted or cell surface proteins [20]. These findings, together with the identification of the novel myoepithelial calcium-binding proteoglycan, SPOCK1, suggest that MECs are actively involved in autocrine–paracrine interactions [21]. Moreover, in the present study, we demonstrated that SPOCK1 enhanced invasion in breast cancer cells and correlated with poor prognosis in breast cancer clinical samples. Intriguingly, SPARC (osteonectin) has also been identified as an independent marker of poor prognosis in breast cancers [7,22]. Together, these observations substantiated the notion that dysregulation of certain MEC markers confers poor prognosis in breast cancers [5–7]. In addition, our finding is consistent with previous studies demonstrating that SPOCK1 plays a critical role in multiple cancers, including prostate cancer, glioblastoma, hepatocellular carcinoma, esophageal squamous cell carcinoma, lung carcinoma, and gall bladder carcinoma [16,19,23–26]. These studies confirm the role of SPOCK1 in the development and progression of multiple cancers and imply a potential role for SPOCK1 as a therapeutic target.

In this study, we identified SPOCK1 as a novel TGF-β–induced MEC marker. TGF-β is a multifunctional protein involving tissue repair, wound healing, and progressive fibrosis [9,27]. In addition to its effect on extracellular matrix turnover, TGF-β is known to affect cell phenotype, including the induction of contractile phenotype and the upregulation of α-smooth muscle actin (α-SMA) during fibroblast–myofibroblast transdifferentiation [12]. Importantly, α-SMA is the most abundant actin isoform in mammary MECs and the contractile activity of MECs requires the expression of α-SMA and appropriate cell–extracellular matrix interaction [1]. Thus, the identification of SPOCK1 as a TGF-β–induced MEC marker further substantiates a role of TGF-β in the function of MEC. Moreover, although an oncogenic role of SPOCK1 has been demonstrated in multiple cancers, very little is known about the pathogenesis of SPOCK1 dysregulation. We demonstrated a novel role of TGF-β in the induction of the MEC marker, SPOCK1, and the finding may suggest that alteration of the TGF-β pathway may be a potential cause of SPOCK1 dysregulation conferring poor prognosis in breast cancer. Moreover, the upregulation of SPOCK1 was observed in lung cancer cell A549 treated with TGF-β [25]. Although SPOCK1 was shown to be a CHD1L-upregulated gene underlying CHD1L-induced hepatocarcinogenesis [16,28], CDH1 expression was not altered in SPOCK1-expressing human breast cells induced by TGF-β (data not shown), suggesting that alteration of CDH1 is not involved in SPOCK1 expression in the breast.

In this study, we investigated the *in situ* expression patterns of the MEC marker, SPOCK1, in 478 IDC cases, as well as in 81 non-IDC histological carcinoma subtypes. We found expression of SPOCK1 in 18.8% of IDC cases, and the expression was significantly correlated with a high histological grade, the triple-negative phenotype, and the basal-like phenotype. Consistent with the association between SPOCK1 expression and such aggressive phenotypes, we have shown a significant correlation between SPOCK1 expression and poorer overall survival in our breast cancer cohort. The expression of SPOCK1 in up to 66.7% of MBC but rarely in other subtypes of breast carcinoma is remarkable. MBC, typically characterized by the coexistence of carcinomatous and sarcomatous components, has been shown to consistently harbor basal/myoepithelial phenotype and may represent morphological spectrum of basal-like and myoepithelial breast carcinomas [28–30]. Accumulating evidence indicates the critical role of epithelial–mesenchymal transition (EMT) in the pathogenesis of MBC [22,28,31,32]. Intriguingly, SPOCK1 expression has been shown to induce EMT in multiple cancer cell lines, including gall bladder, lung, and esophagus, and has been demonstrated to confer resistance to HER2 target therapy in gastric cancer cell line through EMT [24–26,33]. Thus, the enrichment of SPOCK1 expression in MBC and the correlation between SPOCK1 expression and triple-negative or basal-like IDC that have been shown to preferentially express EMT markers [34] provide in vivo evidence for an association between SPOCK1 and the EMT, and support such an association being present in cell lines.

In conclusion, we identified SPOCK1 as a novel TGF-β–induced MEC marker and further demonstrated that SPOCK1 enhanced invasion in breast cancer cells and correlated with poor prognosis in breast cancer clinical samples. Our finding supports the role of certain myoepithelial markers in tumor progression and suggested that the alteration of the TGF-β pathway may be a potential cause of SPOCK1 dysregulation, conferring poor prognosis in breast cancer. The enrichment of SPOCK1 expression in MBC and the correlation between SPOCK1 expression and high histological grading and basal-like phenotypes in IDC evidence an association between SPOCK1 and the EMT.

## Supporting Information

S1 Table Top 30 TGF-β-upregulated genes in MCF10A cells. (DOC)
