# Supplementary material for: SPOCK1 Is a Novel Transforming Growth Factor-β–Induced Myoepithelial Marker That Enhances Invasion and Correlates with Poor Prognosis in Breast Cancer
Source: PLoS One. 2016 Sep 14;11(9):e0162933. doi: 10.1371/journal.pone.0162933 (PMC5023187; doi:10.1371/journal.pone.0162933)
Supplement: S1 Table — (DOC) [file pone.0162933.s001.doc]

**Supporting Table 1. Top 30 TGF-β-upregulated genes in MCF10A cells**

| Gene symbol | Gene name | IHC antibody available^1^ | | IHC antibody not available |
| --- | --- | --- | --- | --- |
|  |  | IHC information not available  IHC performed in this study ^2^ | IHC Information available  IHC not performed in this study^3^ |  |
| PRR5L | proline rich 5 like |  |  | V |
| POSTN | periostin | V |  |  |
| CXCR7 | chemokine (C-X-C motif) receptor 7 | V |  |  |
| IGFL2 | IGF-like family member 2 | V |  |  |
| SPOCK1 | sparc/osteonectin, cwcv and kazal-like domains proteoglycan 1 | V |  |  |
| HS3ST2 | heparan sulfate 3-O-sulfotransferase 2 |  | V |  |
| KCNJ15 | potassium inwardly-rectifying channel, subfamily J, member 15 |  |  | V |
| PDE6A | phosphodiesterase 6A, cGMP-specific, rod, alpha |  |  | V |
| CHRNA9 | cholinergic receptor, nicotinic, alpha 9 |  | V |  |
| PLD5 | phospholipase D family, member 5 |  |  | V |
| PCDH9 | protocadherin 9 | V |  |  |
| PDGFRB | platelet-derived growth factor receptor, beta polypeptide |  | V |  |
| GPAM | ens|glycerol-3-phosphate acyltransferase, mitochondrial |  |  | V |
| ADAMTS4 | ADAM metallopeptidase with thrombospondin type 1 motif, 4 |  |  | V |
| GLI1 | GLI family zinc finger 1 |  | V |  |
| PRRX2 | paired related homeobox 2 | V |  |  |
| ADAMTS6 | ADAM metallopeptidase with thrombospondin type 1 motif, 6 |  |  | V |
| ZEB1 | zinc finger E-box binding homeobox 1 |  | V |  |
| AKNAD1 | AKNA domain containing 1 |  |  | V |
| FN1 | fibronectin 1 |  | V |  |
| VIM | vimentin |  | V |  |
| TGFBI | transforming growth factor, beta-induced |  |  | V |
| FBN1 | fibrillin 1 | V |  |  |
| SCG5 | secretogranin V |  |  | V |
| PLAT | plasminogen activator, tissue | V |  |  |
| PIWIL4 | piwi-like 4 (Drosophila) |  |  | V |
| HAS2 | hyaluronan synthase 2 |  | V |  |
| PAGE3 | P antigen family, member 3 |  |  | V |
| IL11 | interleukin 11 |  |  | V |
| NOV | nephroblastoma overexpressed gene | V |  |  |

IHC, immunohistochemistry

^1^ Antibody suitable for immunohistochemistry on paraffin tissue section is available in the literature.

2 Information about the immunolocalization of the gene products was not available; therefore, immunohistochemistry was performed.

3. Information about the immunolocalization of the gene products was available in the literature; therefore, immunohistochemistry was not performed.
